# Increased CCL19 expression is associated with progression in cervical cancer

**DOI:** 10.18632/oncotarget.17982

**Published:** 2017-05-18

**Authors:** Xiaoshu Zhang, Yue Wang, Yanning Cao, Xueshan Zhang, Haiya Zhao

**Affiliations:** ^1^ Department of Immunology, Binzhou Medical University, Yantai, 264003, China

**Keywords:** cervical cancer, CCL19, EMT, invasion, proliferation

## Abstract

Cervical cancer is the third most common cancer and the fourth leading cause of malignancy related mortality in women worldwide. CCL19 is highly expressed in human cancer cells, and ligand CCL19 binding to CCR7 induces actin polymerization and pseudopodia formation. However, whether or not CCL19 is involved in EMT of human cervical cancer needs further investigation. Using quantitative PCR and western blot analyses, we found that CCL19 is overexpressed in cervical cancer cell lines and tissues. Knockdown of CCL19 via siRNA inhibited the proliferation of cervical cancer cells by increasing apoptosis. Further analyses showed that inhibitory effects of CCL19 on cell migration and invasion were partly associated with EMT process. In conclusion, these data indicate that CCL19 is abnormally expressed in cervical cancer, indicating a novel and important role for CCL19 in cervical cancer malignant transformation.

## INTRODUCTION

Cervical cancer has been generally considered as one of the most common cancers in women worldwide [1]. Certain types of the human papilloma virus (HPV) infection, particularly HPV 16 and HPV 18, are the greatest risk factors for cervical cancer [2]. Despite the great advances achieved in surgical techniques, metastatic and recurrent cervical cancer remains the major causes of cancer-related deaths [3]. Molecular alterations of tumor suppressor genes and/or oncogenes have a pivotal role in the progression of cervical cancer.

Increasing evidences indicate that both chemokines and chemokine receptors play critical roles in progression of solid tumors including cervical cancer [4–5]. Moreover, previous studies show that numbers chemokines (i.e. CCL19, CXCL12) overexpressed in various tumors, which activate tumor proliferation related signaling pathway and stimulate angiogenesis on one hand, recruit immune cells infiltrating into cancerous foci and trigger tumor associated inflammation on the other [6]. The chemokines CCL19 could modulate the inflammatory responses [7]. CCR7, one of G protein-coupled chemokine receptors, lead to alteration of cell skeleton rearrangement and cell migration by binding its ligand CCL19 [8]. Considerable studies have focused on the potential role of CCL19/ CCR7 axis in several cancers [9–11]. However, the role of CCL19 in cervical cancer has not been comprehensively studied until recently. This study highlights the emerging role of CCL19*,* and suggests potential target of CCL19 pathway in cervical cancer tumorigenesis.

## RESULTS

**Figure 1:**
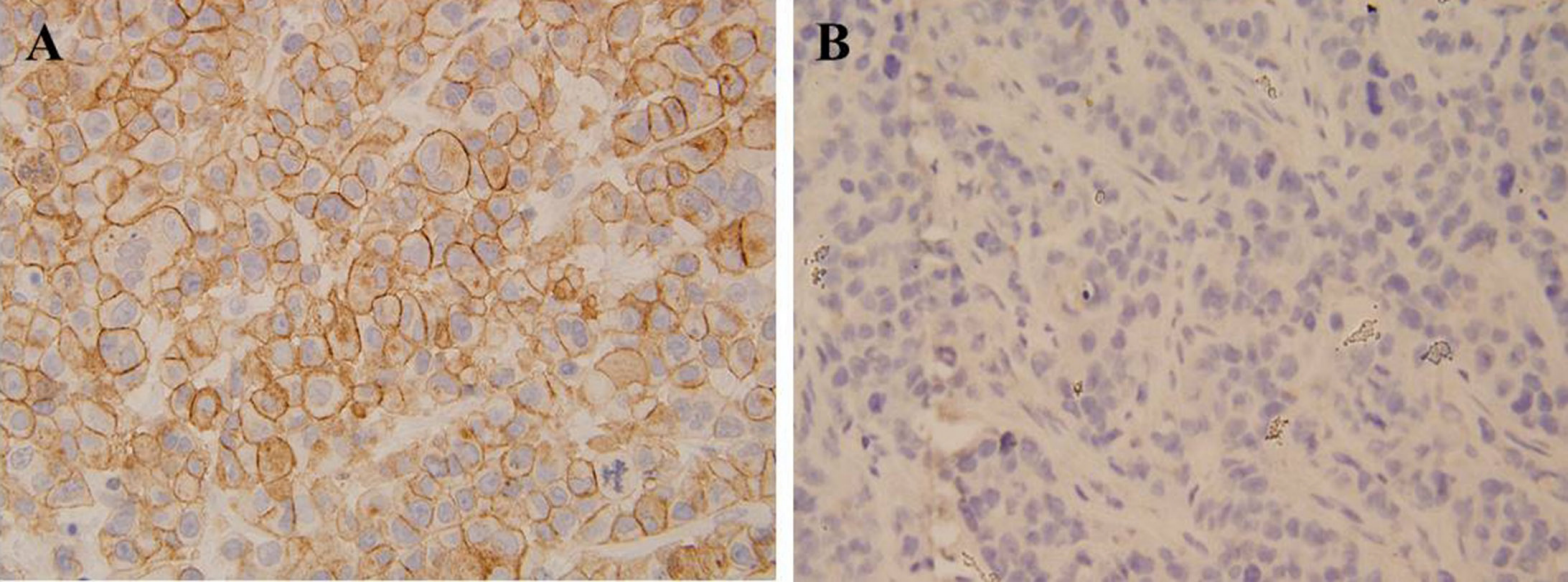
Immunohistochemical analysis revealed that cervical cancer tissue had stronger CCL19 expression compared with that of corresponding adjacent noncancerous tissues

**Figure 2:**
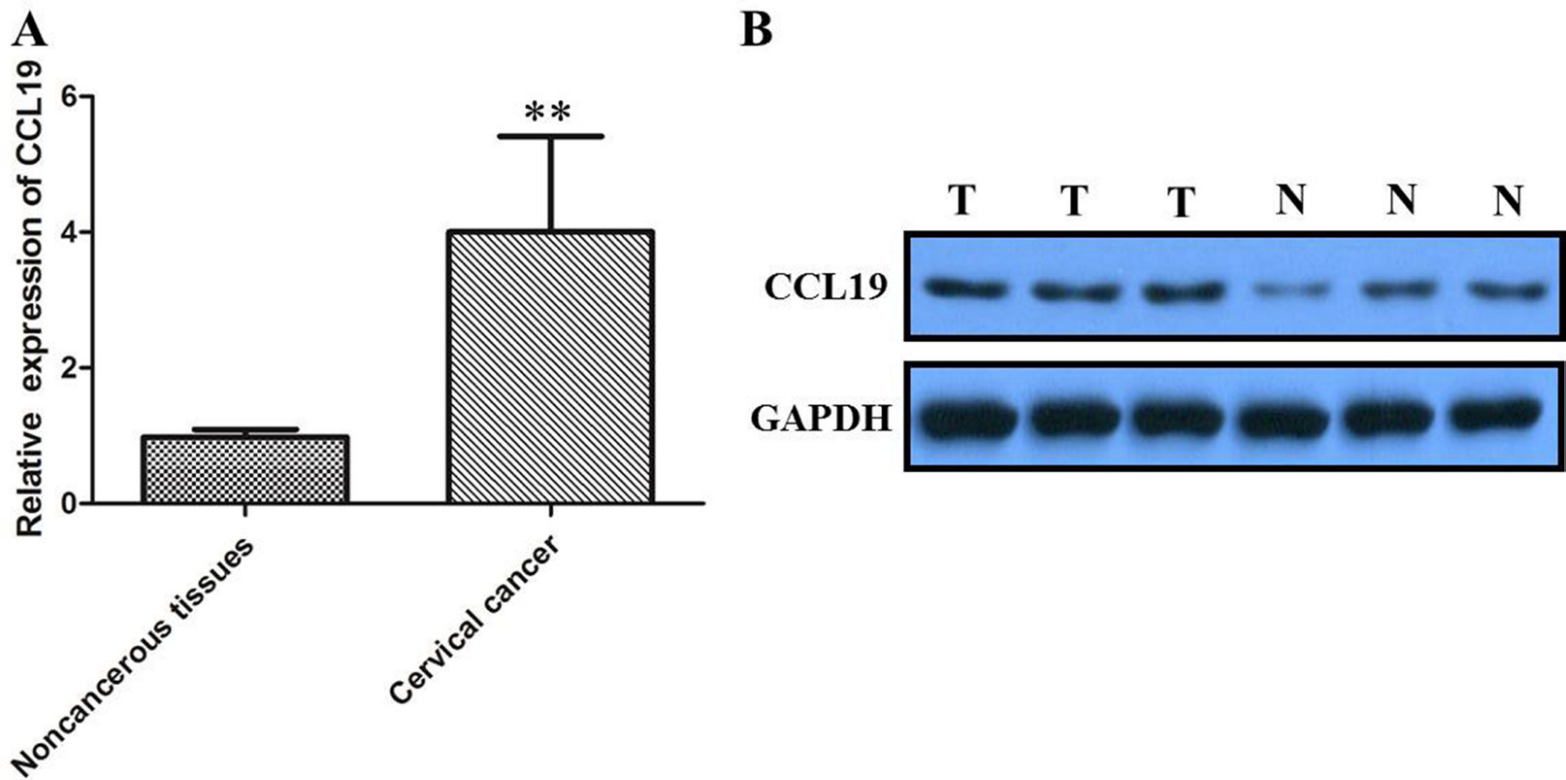
(**A**) qRT-PCR showing expression level of CCL19 mRNA in cervical cancer tissue; (**B**) Western blots showing the expression of CCL191 protein in cervical cancer tissue; *P < 0.05, **P < 0.01.

**Figure 3:**
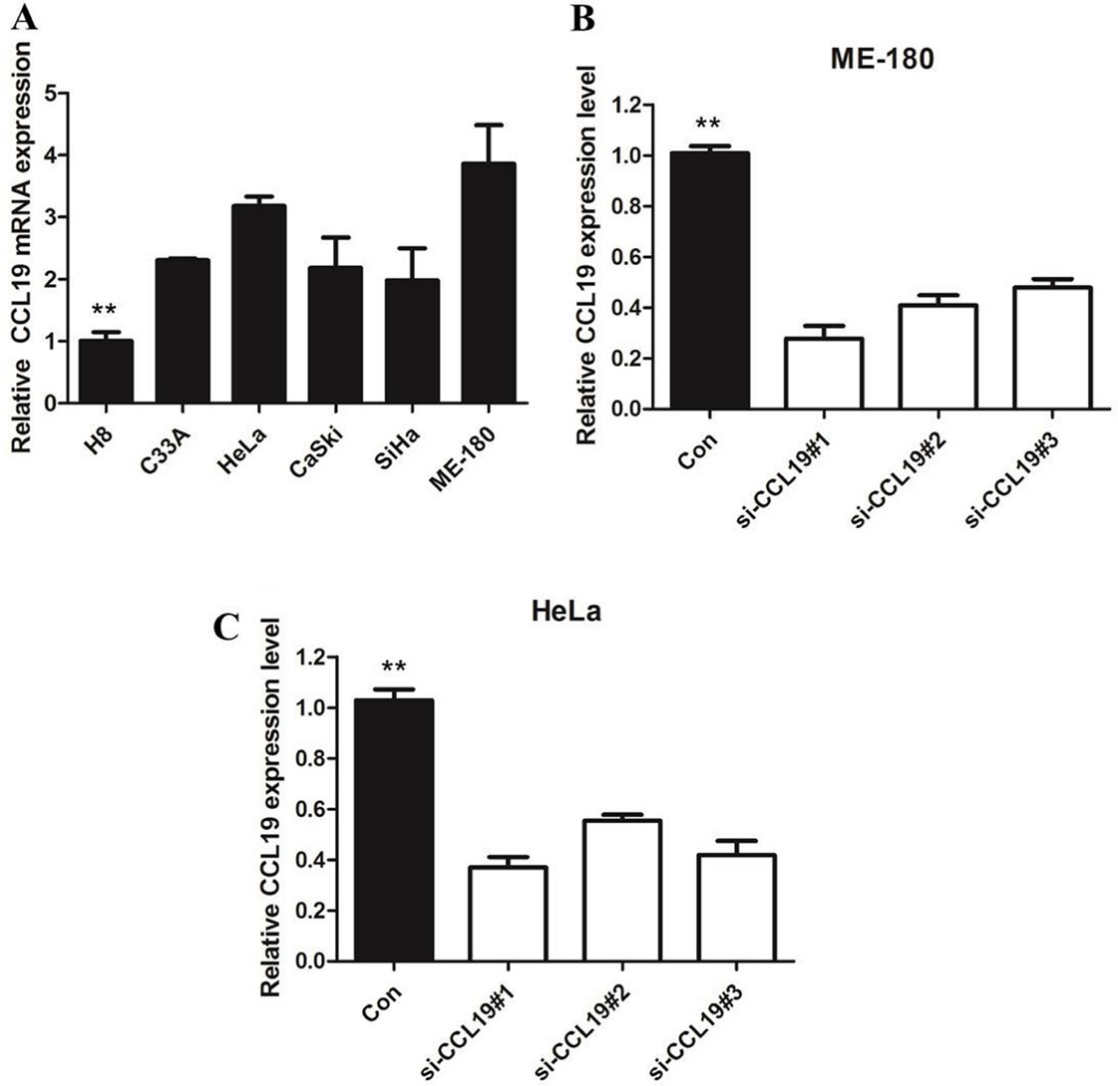
(**A)** qRT-PCR showing expression level of CCL19 mRNA in cervical cancer-derived cell lines; (**B**) qRT-PCR showing the expression of CCL19 mRNA in ME-180/si cells was significantly decreased compared with control cells; (**C**) qRT-PCR showing the expression of CCL19 mRNA in Hela/si cells was significantly decreased compared with control cells; *P < 0.05, **P < 0.01.

Using IHC, the CCL19 expression levels were detected in a total of 62 cervical cancer and adjacent non-cancerous tissues. As shown in Figure 1, CCL19 was significantly overexpressed in tumor tissues (88.71%; 55/62) than in normal counter parts (37.10%; 23/62, *P* < 0.001). Besides, CCL19 expression was positively correlated with the tumor diameter (*p* = 0.001) and TNM stage grouping (*p* = 0.001). CCL19 mRNA and protein expression were significantly higher in tumor group than in noncancerous tissue group (*p* < 0.01; Figure 2A and 2B). CCL19 was higher expressed in cervical cancer cell lines (C33A, HeLa, CaSki, SiHa, and ME-180) than in normal human cervical epithelial cell line H8 (Figure 3A).

In order to investigate the role of CCL19 in cervical cancer cell lines, three siRNA was used to knockdown CCL19 expression in ME-180 and HeLa which have the highest of CCL19 expression (Figure 3B and 3C). Our results showed the si-CCL19#1 is the most efficient one to down-regulate CCL19 expression.

**Figure 4:**
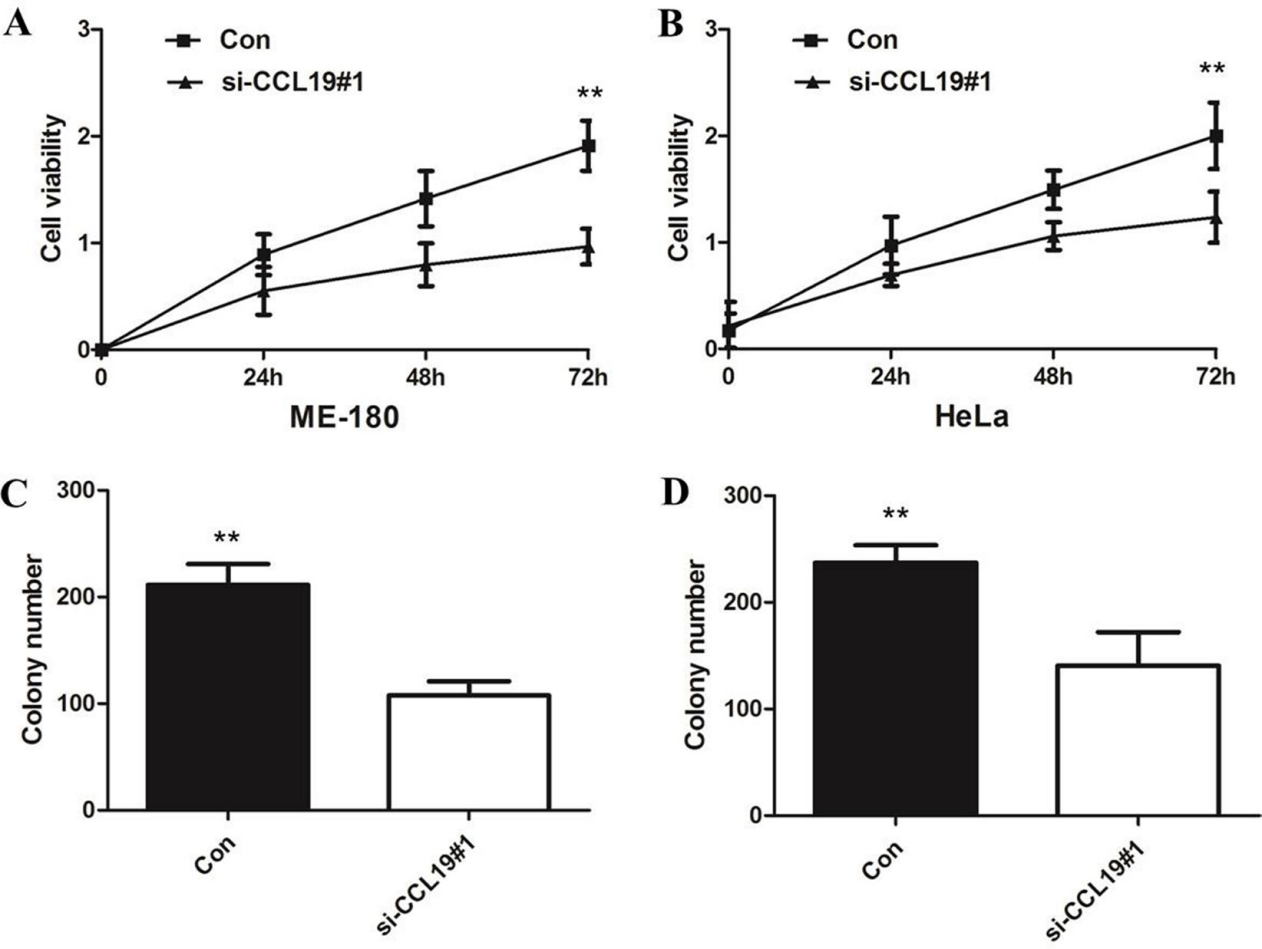
(**A**) CCK8 assay showing knockdown of CCL19 inhibited cell proliferation of ME-180 cells; (**B**) CCK8 assay showing knockdown of CCL19 inhibited cell proliferation of HeLa cells; (**C**) Colony-formation assays showed that silencing of CCL19 significantly increased the colony-forming ability of ME-180 cells; (**D**) Colony-formation assays showed that silencing of CCL19 significantly increased the colony-forming ability of HeLa cells; *P < 0.05, **P < 0.01.

**Figure 5:**
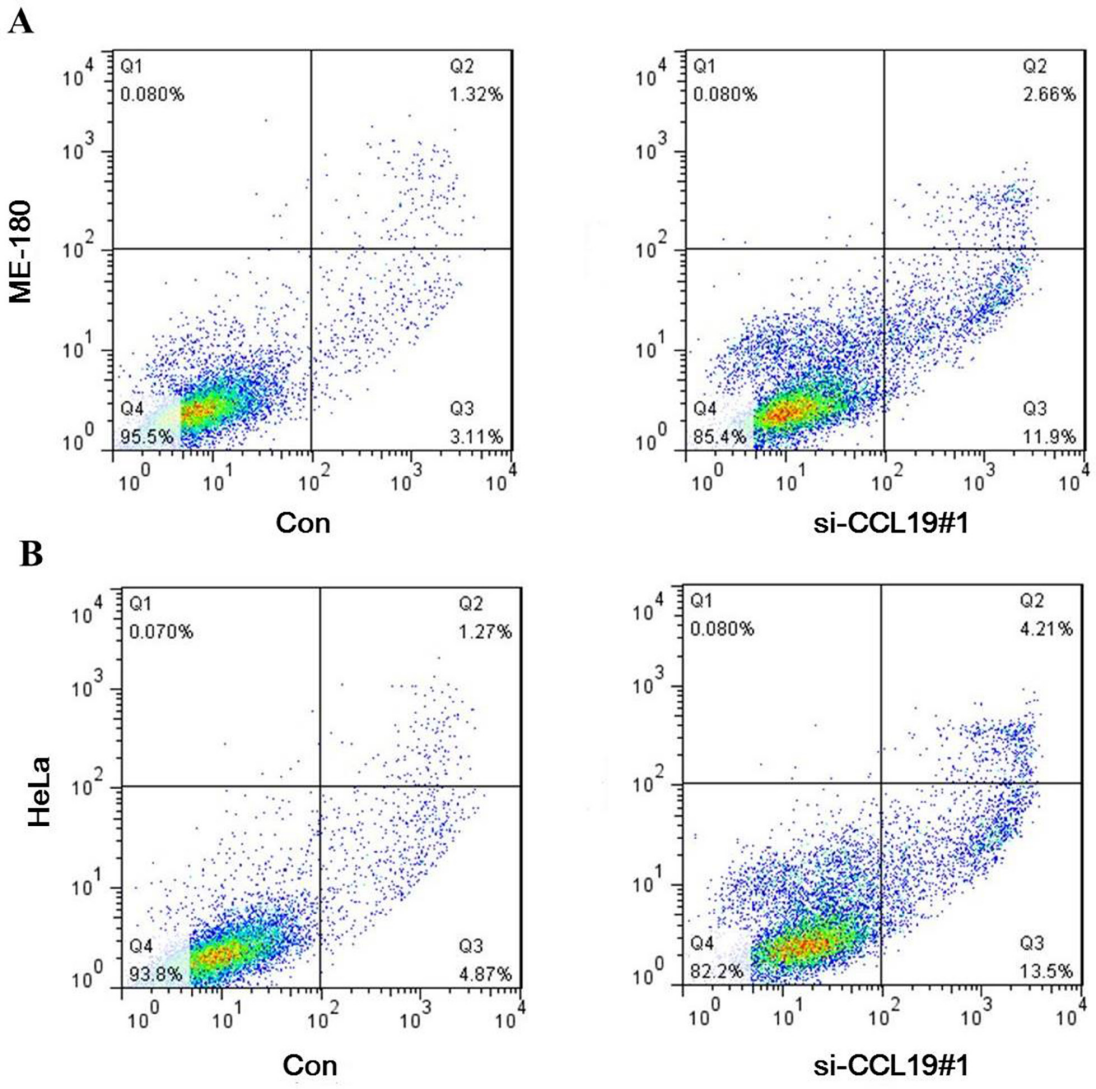
(**A**) Down-regulation of CCL19 expression by siRNA increased apoptosis in ME-180 cells; (**B**) Down-regulation of CCL19 expression by siRNA increased apoptosis in HeLa cells;

**Figure 6:**
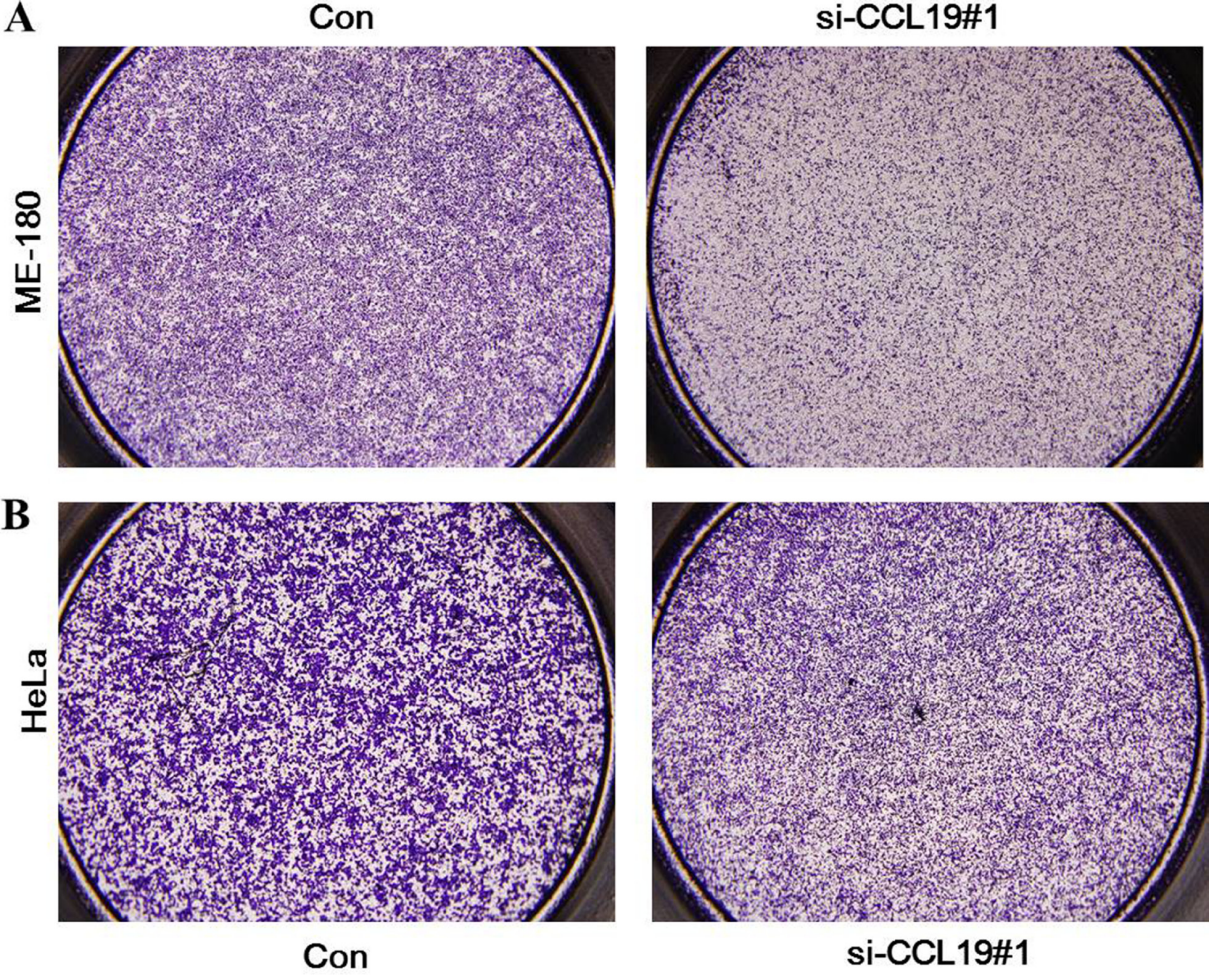
(**A**) Inhibition of Migration and Invasion of ME-180 cells by CCL19 siRNA; (**B**) Inhibition of Migration and Invasion of HeLa cells by CCL19 siRNA;

CCK8 assay showed that knockdown of CCL19 attenuated cell proliferation in ME-180 and HeLa cells. Colony-formation assay showed that clonogenic survival was significantly decreased after the knockdown of CCL19 (*P* < 0.05; Figure 4C and 4D). These data are consistent with the notion that CCL19 is required for the proliferation of cervical cancer cells. Annexin VFITC analysis was used to detect the rate of apoptosis in ME-180 and HeLa cells. The results showed that CCL19 depletion could induce a significant population of early and late apoptotic of ME-180 and HeLa cells compared with controls (Figure 5A and 5B). The results of transwell assays showed that knockdown of CCAT1 significantly repressed the migration and invasive ability of ME-180 and HeLa cells (*p* < 0.001; Figure 6A–6B).

**Figure 7:**
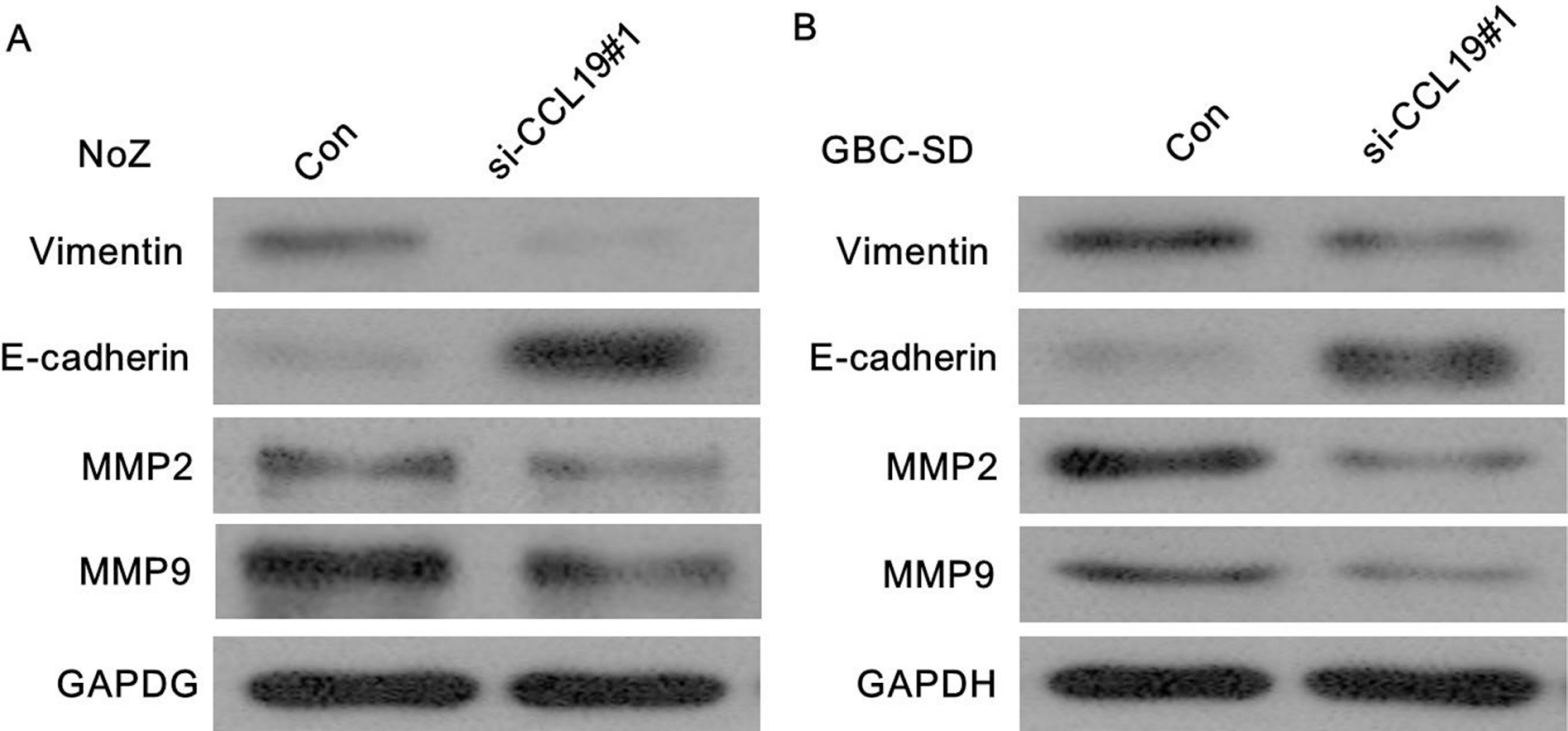
(**A**) Knockdown of CCL19 reverses EMT in ME-180 cells; (**B**) Knockdown of CCL19 reverses EMT in HeLa cells.

Because EMT is the remarkable presentation for cell invasion, whether silencing CCL19 expression inhibited mesenchymal features need to be identified. As showed in Figure 7, Vimentin and N-cadherin were downregulated after CCL19 knockdown while E-cadherin was overexpressed in ME-180 and HeLa cells. Therefore, inhibition of CCL19 in cervical cancer cells makes the cell phenotype be more epithelial rather than mesenchymal. Recent studies suggest that MMP2 and MMP9 modulate cell migration and lineage development. Our results showed that MMP2 and MMP9 were found to be markedly downregulated after CCL19 interference (*P* < 0.001).

## DISCUSSION

Previous studies have revealed the prevalence of cervical HPV infection in cervical cancer varies greatly worldwide [12]. Despite of the recent rapid promotion in the diagnosis and therapy, the prognosis of cervical cancer remains poor because the diagnosis of most of cervical cancer patients are defined at a advanced stage, and patients had lymphatic metastasis of cervical cancer [13]. Increasing evidence have demonstrated that many signaling pathways affected the development of cervical cancer, among the imbalance between oncogenes and tumor suppressor genes is responsible for the development of tumors, however the specific molecular mechanism of cervical cancer is not yet fully elucidated. Understanding its molecular mechanisms may offer the potential for the identification of new prognostic biomarkers and therapeutic targets.

Chemokines could attract and activate cells to specific locations in the body [14–16]. Recent literatures have identified the interaction between CCL19 and human cancer cells [17]. However, whether or not CCL19 is involved in progression of human cervical cancer needs further investigation. In our study, CCL19 was firstly investigated to be upregulated in tumor tissues both at the transcription and protein level in cervical cancer. Moreover, our IHC results showed that the positive rate of CCL19 staining was 88.71% in 62 cases of cervical cancer and 37.10% in 62 cases of peritumoral tissues. Besides, we also found that all cervical cancer-derived cell line expressed higher CCL19 than the normal human cervical epithelial cell line H8, especially for ME-180 and HeLa cells. In fact, CCL19 has already been found to be overexpressed in various carcinomas [18–20].

To further illustrate the role and function of CCL19 in cervical cancer, we knockdowned the CCL19 expression in cervical cancer cell lines ME-180 and HeLa. Our results showed that silencing of CCL19 significantly inhibited cell proliferation, colony formation, migration and invasion of cervical cancer cells. These results demonstrated that CCL19 is associated with malignant transformation and promoted cancer growth in cervical cancer, which is consistent with previous study of CCL19 in other cancer cells [18–20]. Moreover, down-regulation of CCL19 expression induces apoptosis in ME-180 and HeLa cells, indicating that knockdown of CCL19 led to growth inhibition of cervical cancer *in vitro* might be correlated with cell apoptosis enhancement.

EMT is a vital process by which epithelial cells lose polarity and adhesion, and gain migratory and invasive properties, which has been associated with pathogenesis of tremendous cancers, including cervical cancer [21–22]. Moreover, the EMT phenotype was reported to be associated with tumor invasion, carcinoma metastasis, drug resistance and stem cell proportion [23–24]. Here, we confirmed that knockdown of CCL19 suppressed EMT through the downregulation of vimentin, consistent with upregulation of E-cadherin in cervical cancer cells. MMP-2 and MMP-9 are members of metalloproteases family that can degrade extracellular matrix proteins. Previous studies demonstrated that the expression of MMP-2 and MMP-9 is crucial in cervical cancer metastasis [25]. High MMP2 and MMP9 expression are thought to be crucial for the migration and invasion. Our results showed that inhibition of CCL19 inhibited expression of MMP-9 and MMP-2, suggesting that CCL19 increase the aggressiveness of cervical cancer cells, possibly by regulation of MMP-2 and MMP-9.

In summary, the present study revealed that CCL19 was up-regulated in cervical cancer tissues. Besides, our study highlighted the interaction between CCL19 and cervical cancer cell proliferation, migration, invasion. Our findings provide new insights into the function of CCL19 in the development of cervical cancer and suggest that CCL19 might be considered as a potential target for the cervical cancer therapies in the future.

## MATERIALS AND METHODS

### Clinical specimens

A total of 62 cervical cancer tissues and adjacent normal tissues were collected from patients who underwent surgical resection between 2010 and 2014. Clinical information was obtained using the database of patients. None of the patients had received previous radiotherapy, chemotherapy, or other treatments before surgery. The clinical stage was determined by using the International League of Gynecology and Obstetrics (FIGO). After surgical removal, the tissues were frozen immediately in liquid nitrogen until use. The tissues were formalin-fixed and paraffin-embedded for histopathologic diagnosis and immunohistochemical staining.

### Cell culture

Five cervical cancer cell lines (C33A, HeLa, CaSki, SiHa, ME-180) and a normal human cervical epithelial cell line (H8) were maintained at 37°C in an atmosphere of 5% CO_2_ in DMEM with 10% fetal bovine serum.

### Immunohistochemistry

Paraffin-embedded tissue blocks were cut into 2-μm-thick serial sections, deparaffinized in xylene and rehydrated by using descending concentrations of ethanol. After antigen retrieval by microwave heating or pressure cooking, the slides were immersed in 3% hydrogen peroxide to block endogenous peroxidase. Then each section was incubated successively with anti-CCL19 primary antibody (R&D, Minneapolis, MN, USA) at 4°C overnight and the corresponding secondary antibody at 25°C for 30 min. Subsequently, 3,3′-diaminobenzidine and hematoxylin were used for color development and counterstain, respectively. Besides, one section of each block was picked and routinely stained with hematoxylin and eosin for standard pathological diagnosis. All immunohistochemistry and hematoxylin–eosin slides were reviewed by two pathologists to confirm the diagnoses. PBS was used instead of primary antibody for negative control slides, and normal cervical epithelium was employed as the positive control. The staining intensity in epithelial cells was evaluated on the following scale: 0 for a negative stain, 1 for weak positivity, 2 for median positivity and 3 for strong positivity. The area containing positive cells was scored as 0 to 100%. Next, the score was calculated as the intensity of positivity multiplied by the positive area. When the score was < 4, it was negative or low expression; positive or high expression when the score was equal to or more than 4.

### Quantitative real-time PCR

Total RNA was extracted from tissues using TRIzol reagent (Invitrogen, Carlsbad, CA) according to the manufacturer’s protocol. Amplification of cDNA was performed by specific primers. Quantitative real-time PCR was carried out by using SYBR Green PCR master mix (Applied Biosystems) on a system of Rotor-Gene 6000 (QIAGEN). GAPDH mRNA levels were used as internal control. Fold changes were calculated and normalized using the −ΔΔCT method and the mRNA level compared with those of controls. PCR primers were as follows: CCL19 sense primer: 5′-GTGACCTGCATTAACTCTTTACTTGC-3′; antisense primer: 5′-TATGGCTCTGGCTCTACTGGTTG-3′. Glyceraldehyde 3-phosphate dehydrogenase (GAPDH) sense primer: 5′-GGACCTGACCTGCCGTCTAG-3′; antisense primer: 5′-GTAGCCCAGGATGCCCTFGA-3′.

### Western blotting analysis

Total protein from cultured cells was extracted in cell lysis buffer (PIERCE, Rockford, IL) and quantified using the Bradford method. Fifty micrograms of protein were loaded and separated on SDS-PAGE (12%). After transferring to a polyvinylidene fluoride membrane (Millipore, Billerica, MA), the membrane was incubated overnight at 48°C with polyclonal goat anti-human CCL19 (N-18; sc-9777, Santa Cruz Biotechnology, Inc., Santa Cruz, CA, USA, 1:100) and mouse polyclonal anti-GAPDH antibody (Santa Cruz Biotechnology; 1:1,000). After incubation with peroxidase-conjugated anti-mouse IgG (Santa Cruz Biotechnology) at 37°C for 2 h, bound proteins were visualized using ECL (Pierce) and detected using BioImaging Systems (UVP Inc., Upland, CA). The relative protein levels were calculated by normalizing to β-actin protein as a loading reference.

### Transfection

Cells were plated in six-well plates. Twenty-four hours later, the cells were transfected with control siRNA or with CCL19 siRNA (Santa Cruz, CA, USA) using siRNA transfection reagent (Santa Cruz Biotechnology, Inc.) according to the manufacturer’s instructions.

### Cell proliferation assay

Cell proliferation was measured using the cell counting kit-8 (CCK-8) (Dōjindo Laboratories, Shanghai, China). Cells were seeded at a final concentration of 4 × 10^4^ cells/well and cultured in 96-well flat-bottomed microplates. Then, CCK-8 reagent (10 μL) was added to each well containing 100 μL of culture medium, and the plates were incubated for 2 h at 37°C. Viable cells were evaluated by absorbance measurements at 450 nm using an auto microplate reader. OD_450_ was proportional to the degree of cell proliferation. All experiments were performed five times in three independent experimental trials.

### Colony formation assay

Cells were plated into three 6-cm cell culture dishes after transfected with siRNA or control siRNA. Cells were incubated for two weeks in complete growth media. Cell colonies were fixed with cold methanol stained with 0.1% crystal violet for 30 min. The colonies were manually counted using a microscope.

### Apoptosis analysis

At transfection, cells were harvested and washed with ice-cold phosphate-buffered saline twice. Then, cells were re-suspended in annexin v-binding buffer and the indicated amount of propidium iodide and annexinV-FITC (BD Pharmingen, San Diego, CA, USA) was added. Cells were analyzed by flow cytometry (Calibur, BD, USA). The proportion of apoptotic cells (Annexin-V positive cells) were shown as the mean ± SD.

### Transwell migration assays

Tumor cell *in vitro* migration assays was carried out using Transwell system with polyethylene terephthalate membrane (24-well inserts, 8.0µm; Corning). After transfection, tumor cells were detached and re-suspended in serum free medium. Cells were added to the top champers. Complete growth medium was added to the bottom wells as migration stimulation. After incubation for 18 hours, cells on the membrane of top chambers were fixed with 90% ethanol and subsequently stained with 0.1% crystal violet. Quantification was performed by measuring OD value at a wavelength of 570 nm.

### Statistical analysis

Data analyses were performed using SPSS statistical package 15.0. Patient characteristics are shown as the mean ± SD for continuous variables, and as the count and percent for discrete variables. Phenotypic differences in quantitative traits were assessed by genotype using the *t* test or ANOVA. Differences in the distribution of qualitative traits by genotype were assessed by standard chi-square analysis and Fisher’s exact test. A *P* value less than 0.05 was considered significant.
